# Cardiovascular Disease in the Peruvian Andes: Local Perceptions, Barriers, and Paths to Preventing Chronic Diseases in the Cajamarca Region

**DOI:** 10.3389/ijph.2021.1604117

**Published:** 2021-09-27

**Authors:** Giuliana Sanchez-Samaniego, Stella M. Hartinger, Paula S. Tallman, Daniel Mäusezahl

**Affiliations:** ^1^ Department of Epidemiology and Public Health, Swiss Tropical and Public Health Institute (Swiss TPH), Basel, Switzerland; ^2^ University of Basel, Basel, Switzerland; ^3^ Faculty of Public Health and Administration, Universidad Peruana Cayetano Heredia, Lima, Peru; ^4^ Field Museum of Natural History, Chicago, IL, United States; ^5^ Department of Anthropology, Loyola University Chicago, Chicago, IL, United States

**Keywords:** prevention, cardiovascular disease, Andes, health system, nutrition, Peru, focus groups

## Abstract

**Objectives:** Public health interventions can be improved by understanding peoples’ explanatory models of disease. We explore awareness and perceptions of cardiovascular diseases (CVD) and options for preventative actions in young adults living in rural Andean communities.

**Methods:** We used convenience sampling to select 46 men and women from communities in Cajamarca (Peru). Subjects participated in eight focus groups where they discussed their understanding and perceived causes of CVD as well as barriers and pathways to healthy lifestyles.

**Results:** Fresh foods, physical activity, unpleasant emotions, and healthcare access were cited as important determinants of healthy lifestyles. Barriers to healthy diets included lacking nutritional knowledge, fluctuating food prices, and limited access to foodstuffs. Women felt particularly vulnerable to CVD and identified gendered barriers to manage stress and engage in sports. Low health literacy, poor doctor-patient relationships, and long distances prevented participants from fully accessing healthcare.

**Conclusion:** CVD prevention interventions should consider local knowledge of these diseases and of healthy lifestyles, and harness ongoing programmes that have successfully promoted good nutrition in children and pregnant women. In concert with public-private parterships, governments should include disease prevention interventions for the entire family.

## Introduction

Cardiovascular diseases (CVD) accounted for 20% of deaths in Peru in 2016 [1]. Prevention and control of CVD is a particular challenge in the country, where more than 60% of the population is overweight [2]. Although rates of CVD are higher in the coastal region, Andean communities also have a substantial disease burden [2–5]. Differences in the prevalence of CVD between these regions could be attributed to differing levels of urbanisation [6, 7], which can lead to changes in diet and physical activity and the adoption of unhealthy behaviours such as tobacco use and excessive alcohol consumption [8].

In 2004, the first National Health Strategy for Control and Prevention of Non-Communicable Diseases was published in Peru [9]. However, most of the preventative programming has been undertaken in larger cities and hospitals level, leaving out primary health care in rural areas. To successfully move forward implementing disease prevention programmes in rural Peruvian communities, researchers and practitioners need to understand the locally relevant factors that limit the adoption of healthy behaviours and prevent comprehensive access to healthcare [10].

To address this issue, this study examined local understandings of CVD and healthy lifestyles among Andean adults in Cajamarca, Peru. Specifically, we explored participants’ personal awareness of CVD and the barriers to adopting healthy behaviours using the Health Belief Model (HBM). The HBM framework focuses on understanding personal attitudes, beliefs and practices, which is crucial for the development of intervention approaches that are culturally acceptable, people-driven and bring people closer to the health system [11–13].

## Methods

### Study Setting

This study was carried out in the provinces of San Marcos and Cajabamba in the Cajamarca region, in the northern highlands of Peru between 1800 and 3,900 m above sea level (MASL) [14]. These provinces include peri-urban and rural communities. The majority of the population lives in houses made of clay-sand walls and earthen floors. Farming and livestock are the principal sources of income, although some people also work in the sectors of education, health, construction, municipality, and mining [14]. The diet in these areas is primarily comprised of starchy foods, such as potatoes, wheat, rice, and other tubers. Additionally, some food preparations include small portions of vegetables and meat (mostly pig) or eggs. Local crops include wheat and potatoe varieties, and fruits such as avocado, papaya, cactus, and mandarin grow in the low altitude valley (1800–2000 MASL). Other vegetables, protein sources and non-perishable foods are purchased in the local shops or in the weekly district market [15]. These are typical characteristics for the northern Peruvian Andes, where few communities live over 4000 MASL comprising nonetheless more than two million habitants [16].

Several governmental programmes are active in this area. Nutrition interventions include Vaso de Leche, which gives breakfast to pregnant women and children under 6-years-old and Qaliwarma, which gives breakfast and lunch to school aged children. People can also access conditional cash transfer programmes, such as Juntos which is intended for pregnant women and families with children under 15 years old, and Pension 65 for adults over 65 years old that live in extreme poverty [17]. In the case of Juntos parents must commit to attending their children’s health evaluations and to sending their children to school.

Screening for CVD risk factors, including blood pressure, lipids, and glucose measurements, is done at the health centre level, but it is not extended to the primary health care level in the local health posts. The screening in the San Marcos Health Centre reaches 5–7% of the population, which does not reach the Ministry of Health’s goal of 10% (Personal communication from the San Marcos Health Centre’s Epidemiology office).

### Study Design and Participants

We conducted focus group discussions with adults in the Province of San Marcos and Cajabamba. This research was conducted within the framework of a randomised control trial (RCT) focusing on child health outcomes [14]. A subsample of the parents enrolled in the RCT underwent a metabolic syndrome examination. These participants were distributed across 68 communities [15].

For this qualitative study, we used convenience sampling to select participants from the larger RCT. Subjects from eight out of the 68 potential communities were invited to participate. Communities were located at different altitudes and distances to the main district (Table 1).

**TABLE 1:** Characteristics of focus groups conducted in the Provinces of San Marcos and Cajabamba, Peru 2017.

Characteristics of focus group	Number of women	Number of men	Altitude of community (MASL)	Time to main district^a^ (minutes)
Focus group 1	4	1	2000	50
Focus group 2	5	0	2100	45
Focus group 3	5	1	2030	45
Focus group 4	4	1	2050	45
Focus group 5	6	2	2750	60
Focus group 6	5	2	2600	30
Focus group 7	4	2	3850	120
Focus group 8	4	0	2720	40
Total	37	9	—	—

MASL: metres above sea level.

a Travel distance by car in minutes.

The field supervisor and a fieldworker visited the participants’ household and invited them to take part in the focus groups 3–4 days before the scheduled session. Between 5 and 8 male and female individuals participated in eight focus groups. Two focus groups were comprised of only women, the other seven included the participation of at least one man (Table 1).

### Focus Group Discussions

Focus groups (FG) were held at a central location such as a school or community centre, or at a participant’s house. During the focus group, the first author acted as the moderator, and two assistants worked as observers. The moderator facilitated the discussion and encouraged all participants to discuss the questions included in the FG discussion guide (Supplementary Appendix S1).

The Health Belief Model (HBM) is used to study individual health perceptions and is suitable for identifying factors relevant for the prevention of public health problems such as CVD [11, 12, 18]. This model explores people’s motivations for adopting health behaviours and specifically asks for their feelings and concerns about getting a CVD and its consequences [19]. Additionally, it identifies barriers and enablers of behaviour change. Thus, the HBM may help to explain how to address behaviour change that can improve cardiovascular health. The FG discussion guide was based on the six constructs of the HBM; perceived susceptibility, perceived severity, perceived benefits, perceived barriers, cues to action, and self-efficacy. However, since CVD prevention campaigns are not common in the area and the concepts of CVD were not well known in local communities, we complemented this framework by adding three sections on patterns of distress, perceived causes of CVD, and gendered differences in CVD risk. The latter was added due to the higher prevalence of metabolic syndrome among women in contrast to men [15].

### Data Analysis

We recorded all FG discussions and two assistants took notes on verbal communication. Following each session, project staff transcribed the audio recordings, identified the participant speaking, and re-listened to the tapes to ensure accuracy. Data was organised using the Dedoose qualitative analysis software [20].

For analysis, the codebook consisted of topics identified in the FG guidelines and emergent themes from the data. The moderator and the two observers coded the data. If differences in coding appeared, consensus was reached after a team discussion. Extracts were organised into tables, with identifiers for the focus group, the participant number, and the theme code. As focus groups included communities of different altitudes, which may influence their access to markets and healthcare, we added an identifier next to each quote “high-altitude” (> 2500 MASL) vs. “low-valley” (< 2500MASL). Additional demographic and health information for the participants was available from the larger study where this research was embedded [14, 15].

## Results

### Characteristics of Participants

A total of 46 participants (37 women and 9 men) partook in the eight FGs. Two focus groups lasted 30 min and the other six lasted 1 h on average. This excluded the time needed to explain the project and obtain consent. Demographic and health information of the participants are shown in Table 2.

**TABLE 2:** Demographic and health characteristics of young adult participants of the Provinces of San Marcos and Cajabamba, Peru 2017.

	Women		Men	
	N	Mean ± SD or N (%)	N	Mean ± SD or N (%)
Age (years)	37	29.1 ± 6.0	9	29.3 ± 4.0
Complete secondary	34	9 (27%)	6	4 (67%)
Work in the agriculture sector	34	23 (68%)	6	6 (100%)
Housewife	34	6 (18%)		
Living over 2500 MASL^a^	37	15 (41%)	9	6 (67%)
Body mass index	34		6	
Underweight		0		0
Normal weight		15		3
Overweight		13		3
Obese		6		0
Metabolic syndrome	34	10 (29%)	6	3 (50%)

SD: standard deviation, MASL: metres above sea level.

a We used the 2500 MASL cut off point to define populations living at high altitude [15].

### Main Findings

#### Understandings of CVD

In all FGs at least one participant knew a person who suffered from a CVD, but there was dissatisfaction with the knowledge that was available and accessible about the prevention and causes of CVD. Following up on why there is a lack of information, a 26-year-old woman expanded that she had not received information on CVDs from the health services, “Because the health staff in this health post only cares about the main reason of your visit. For example, child growth control. They just examine that and it’s done” (low-valley). Another 34-year-old man expressed a similar sentiment, “About nutrition, about other diseases, for example, tuberculosis, human immunodeficiency virus infection, cancer. For all those … yes, there is prevention … different pamphlets, but very few about the heart” (high-altitude). High levels of illiteracy are an additional factor complicating understandings of CVD. As a woman in her mid-30 s stated, “The one who knows how to read, can read. But the one who doesn’t know, only observes” (high-altitude).

While the participants expressed dissatisfaction with the level of information available for CVD and cited that they have not had in-depth discussions of CVD previously, they still expressed knowledge about the causes and symptoms of CVD. Specifically, they mentioned that symptoms of CVD included agitation, pain, weakness, and strong beating of the heart and that the ultimate consequences of CVD were heart attacks and death. In all focus groups the participants stated that anyone could develop a heart disease, no matter their weight or age.

Additionally, participants identified a number of drivers of CVD, which fell into four general categories: nutrition, physical activity, emotions, and access to healthcare. We found few differences in the perceived drivers of CVD between the low-valley and high altitude communities. In the case of access to healthy nutrition and access to healthcare, distance to markets and healthcare were important limitations for communities at high altitudes. However, the availability of various foodstuffs in the local and district markets, and the availability and acceptability of health professionals at the health establishments were cited as important drivers of health in both settings.

#### Nutrition

Study participants expressed that CVD is associated with being both over and under-nutrition. Interestingly, a 33-year-old woman stated, “When we have fat in the heart too, and is not necessary that we are fat” (low-valley). Another woman in her mid-30s clearly articulated, “If you are overweight or you are underweight, it is equally bad.” (high-altitude).

Participants also made direct connections between nutritional health and food. A woman in her late-20 s living in the low-valley confided, “Sometimes, we only eat to fill our belly, without realising that it is not truly nourishing us.” The same opinion was shared by a woman in her early-30 s living in a high altitude community who stated that, “Sometimes we eat things that are bad for us and we know that they are going to be bad for us. But we eat out of necessity because there is no other thing to eat.”

The identification of structural barriers to adopting a nutritious diet were common and included not being able to access healthy foods, not having the economic resources to purchase them, and lacking knowledge about how to prepare healthy foods. These barriers varied across geography and occupation. More than one third of the participants were farmers, but reported that they did not regularly consume products from their crops. A man in his mid-30 s told us that, “50% (of all goods) you have to buy and another 50% is produced in the field” (high-altitude).

Participants living in the low altitude valley (2000–2100 MASL) reported that they can grow all types of food. However, community members in higher altitudes (over 2500 MASL) are limited in their crop variety and also had issues with accessing fresh food products such as vegetables, fruits, and animal protein. Barriers to obtaining these products included the cost to reach a market (which could be between 30 min and 2 h away), time spent getting there, and issues with food storage. As a woman in her late-20 s pointed out, “Food cannot be stored for a long time, maybe two or 3 days because we do not have a refrigerator, a place where to keep the food” (high-altitude).

On the other hand, local bodegas provide constant access to non-perishable foods and drinks, which are cheaper. As a 36-year-old man quipped, “Mostly you decide what food to buy according to the economy, right?” (high-altitude). But even buying foods at the bodegas was complicated because food prices changed every week because of the many intermediaries involved. As a woman in her mid-30 s explained, “You know the intermediaries always have to take advantage, right? They live off that, so sometimes they sell a product at a certain price and the next week they change it. Then, in the local store, items are more expensive” (low-valley). Local challenges to accessing, purchasing, and storing foods lead people to preferentially buy non-perishable foods such as rice, noodles, oil, and sugar, especially those participants living in distant communities.

#### Physical Activity

Participants indicated that physical activity was beneficial for their wellbeing and recognised it as a preventive measure for developing CVD. They mentioned being highly active because of their daily agricultural and farming work, which they enjoyed and kept them busy. For example, a man in his late-20 s stated that, “We get health benefits, and economic benefits…. to be useful (for field work)” (high-altitude). However, participants explained that recreational physical activities such as sports were often limited due to lack of time, religious and local beliefs, and gendered factors. A 37-year-old man shed light on this saying, “The word of God (local church) has forbidden us to play football. We always bet sometimes, right? Sometimes we have anger at the partner because he is beating us. Sometimes we do not play well.” (low-valley).

Women appear to face dual pressures. On the one hand, participation in sport appears to be expected of mothers. A mother in her late-20 s expanded by saying, “Sometimes in the schools, in kindergartens, the moms have to participate. There is no way you do not participate. If the babies see that their mother does not want to, then they will not want too. So, you have to participate, no matter what.” (low-valley). Yet there is also gendered pressure to refrain from sports as a 36-year-old woman stated that “Sometimes women are expected to stay at home and take care of their family and not spend leisure time in sports” (high-altitude). Gendered dynamics in CVD risk factors were also evident in group discussions regarding the perceived role of emotional stress in CVD.

#### Negative Emotions

Participants articulated that negative emotions could contribute to heart problems and also that knowing they have health problems could produce negative emotions. A 28-year-old woman stated that, “My aunt … sometimes she suffers attacks. She has been told to avoid being melancholic, to avoid worries. Nothing, nothing. No worries, because if she is worried or melancholy she can die” (low-valley). Indeed, participants connected both physical and emotional stress to strain on the heart. A young woman explained that, “Sometimes when you lose your patience. You feel that your heart beats faster at times like this. Sometimes you have anger, let’s say, it’s a moment … ” (high-altitude), while a woman in her late 30 s expressed a similar sentiment in relation to physical activity, saying, “When you walk and feel agitated, then you feel pain and sometimes “stitches.” Well then, you think it is the heart, right?” (low-valley).

These quotes indicate that negative emotions or over-exertion create heart problems, but participants also indicated that simply knowing about having heart problems can produce substantial distress. Indeed, a number of participants expressed that they would feel sad and worried if they were diagnosed with any CVD as the costs of treatment, or losses due to death, could drive economic insecurity for the entire household. Women worried about themselves and their partners saying, “If my husband gets sick, I have to spend money, I do worry” (high-altitude).

Discussions in the FGs revealed why women appeared to be more prone to this type of worrying. A woman in her late-20 s explained, “It seems to me, I say it is because men go to the farm, they occupy themselves there, they forget about everything. On the other hand, the women stay at home and sometimes we have worries about the children … everything, that can also be it (a risk factor of CVD) or maybe not” (low-valley). A man in his late-30 s expressed a similar explanation, “Maybe … (Women are at higher risk) because sometimes we, men, go to work, it is a single job and the ladies stay at home, they work different tasks … they worry sometimes and think “There are things that I don’t have …. I do not have money” (low-valley)*.*

Finally, participants expressed that they would rather not know about a potential cardiovascular condition because of the stress it might cause, saying, “It is better to not know (that you have a CVD). Just stay quite during the pain, then it goes away and you forget…. if you know what you have, it is worse” (low-valley). However, even for those who are interested in knowing about their CVD risk, there are substantial barriers to accessing knowledge, affordable treatment, and care at the local health posts.

#### Healthcare

Participants cited costs associated with transport and food as a barrier to healthcare. As a result, they stated that they typically only went to the clinic for a pressing health issue and not for regular check-ups. A woman in her late-20 s stated, “If it does not hurt, I don’t go to the doctor. If it hurts, I will go to check what is going on. On the contrary, we should have general check-ups, for example, annually” (high-altitude). Appointments at the health post also needed to be scheduled in person, potentially creating two lengthy and costly trips, and creating a high initial barrier to seek care.

In addition to challenges in accessing care, participants criticised the quality of patient-provider relationships. They felt that they were not treated well nor receiving proper attention because the health professionals were inexperienced and prescribed painkillers for every condition. According to a 27-year-old woman, previously, “There was a good nurse, but they relocated her to the hospital. And here, we only have trainees. This is what happens here at the health post.” Finally, she added “All the time we receive the same medicines. When we go with our children, we already know what they will prescribe to us” (low-valley)*.*

More problematically, participants were receiving conflicting medical advice that was not evidence-based. A 37-year-old women revealed that:

“I went to the health post with my results (lipids and glucose measurements). They (health professional) told me: “No, there is no medicine, you can only take warm water before eating.” I told them I couldn’t eat in the morning. They told me that anyway I should eat a little bit …. but I should eat. They told me to start eating less but not to stop eating. They told me better to buy wine and drink a glass before going to sleep to get rid of the fat. But, I have not seen any results” (low-valley).

This type of medical advice appears to be intersecting with local explanatory models for the treatment of CVD. One 32-year-old woman stated, “For example, if the heart is not well and they (health professional) tell you it is because of the fat then you try to eat things that do not affect your heart. Right? I know that you have to drink pineapple juice and because you know this is good for the heart then you drink it this is a medicine…. right? (low-valley)*.* A similar sentiment was expressed by a 27-year-old woman, “Yes, sometimes one feels bad. You can try to look for flowers, and drink flower waters, you can use also lemon, orange, custard apple” (high-altitude)*.*

It is unclear whether these remedies were coming from healthcare practitioners, traditional healers, or fellow community members. What is apparent is that the public health messaging for preventing and treating CVD is not clear. Despite this, participants related CVD to nutrition and exercise and many participants showed an interest in adopting healthier diets and increasing physical exercise.

## Discussion

In this paper, we explored how Andean adults in the Cajamarca region of Peru understood CVD, including the drivers of CVD risk, and perceived barriers to adopting healthy lifestyles. While the understandings of CVD were basic, study participants stated that nutrition, physical activity, negative emotions, and healthcare were important drivers of risk. Although focus groups were located at different altitudes, with differing physical access to markets and health establishments, all participants shared concerns regarding the availability of food products in markets and the capacity and availability of health professionals. We expand on these findings with particular attention to how they can inform National Health Strategy for Control and Prevention of Non-Communicable Diseases and their strategies for vulnerable high-altitude populations [9].

Many of the participants in this study knew that CVD was potentially life-threatening and expressed a desire to be better informed. However, participants made it clear that health communication strategies via text or print-outs would not reach illiterate community members. Instead, they suggested that nutritional workshops with demonstrations of healthy cooking and nutritional discussion groups could effectively engage community members.

Such nutritional workshops, if designed holistically, could address many of the barriers to the adoption of healthy eating behaviours [21, 22]. Participants understood that healthy diets mean eating various types of food in proper amounts but felt their ability to adopt these diets was limited because they did not know how to prepare healthy meals, and could not afford and access diverse food items. While there are existing programmes providing food to children and pregnant women in these areas, including Vaso de Leche and Qaliwarma, adults without children are notably excluded. As primary programme targets, women have to comply with regular maternal and child health checkups and ensure children’s school attendance [17], making them central stakeholders for such programmes and a regular point of contact for the health system. As primary knowledge-bearers and with their expressed desire for nutritional skill-building, women are the key health information channels into the family and are ideal partners for people-centred engagement in CVD prevention in remote Andean communities.

We suggest that future studies should be conducted in close collaboration with Andean women to design nutritional interventions that can improve health and wellbeing at all life stages. There are existing governmental programmes such as Haku Wiñay (“My enterprising farm” in Quechua language), which supports the raising and trading of domestic animals and entrepreneurship in small-scale agriculture [17]. However, this programme started as a pilot in 2014 and is still under development. The programme increased participants well-being and food security but did not necessarily increase their economic income, which is one of its main objectives [23]. Programme evaluators identified also a lack of attention to gendered issues and suggested that women should be involved as key stakeholders in the future.

Additionally, opportunities exist to build on public-private partnerships for CVD prevention. For example, the Peruvian national dairy company collects milk from the remotest areas of the Andes at least weekly. Considering that the company maintains this regular supply chain with remote outreach, building public-private partnerships could help in providing healthy foods to highlanders who otherwise travel only monthly or bi-weekly to local markets. However, to be feasible, such initiatives need to be linked to system-wide changes and interventions.

Peru has successfully executed multi-level actions to address childhood undernutrition and anemia using a combination of programmes in the areas of health, education, cash transfer, water and sanitation, housing, and agriculture [24, 25]. CVD prevention action would need the same approach and could build on such ongoing programmes that have established contacts within the health system. Specifically, CVD risk needs to be assessed at the peripheral primary care level through measurements of anthropometrics and blood pressure, and via short risk questionnaires [26, 27]. Health data could then be saved in a national repository of chronic conditions similar to the Wawared system for pregnant women and children [28]. With surveillance systems in place, monitoring, evaluation and access to treatment can become feasible in distant populations.

Task-shifting [29] is another option that has contributed to maternal and child health programmes in Peru in the past and could be utilised for CVD interventions. Task-shifting entails working with community health workers (CHW) to bring preventative strategies to the periphery and is potentially helpful in settings with health workforce shortages [30]. Task-shifting requires the training of CHW in new skills, the use of standardised protocols, and the provision of adequate equipment for CVD prevention and communication. In this way, health professionals can expand from a child-centred health approach to include adult health. Initial research shows that CHW can be effectively trained in prevention and management guidelines of CVD [31]. The above recommendations are inherently people-centred and community-based and can potentially be the first steps to create more comprehensive national guidelines for CVD prevention in Peru [9].

This study has a number of limitations, including that it was restricted to the northern Andes of Peru, used convenience sampling, and was conducted in young adults in their 30 s. Perceptions of CVD may differ in other Andean areas and in older individuals. Villagers participating in the FG also knew each other, potentially influencing their participation. However, they collectively reflected a community view, which was particularly valuable when discussing locally feasible and acceptable intervention approaches. Additionally, most of the FG participants were women, which may have biased the answers especially about gendered differences. However, as household managers and family-health knowledge-bearers with the most regular contact with health facilities, we believe they and their participating husbands closely represent the broader community perspective on CVD health matters. Lastly, sometimes participants answered our questions but referred to other disease experiences such as malnutrition and infection. Adding the sections of patterns of distress and perceived symptoms to the HBM helped in the understanding of our questions.

In conclusion, this research revealed that there are many feasible entry points of intervention that can prevent Andean community members from succumbing to world-wide increases in CVD. Specifically, local women are key stakeholders who can influence their own health and the health of all family members. Therefore, they must be empowered with the knowledge and resources to enact healthy lifestyles. We see substantial opportunities for this to occur by expanding existing government programmes that are addressing child nutrition to make healthy foods and healthcare accessible, promoting healthy lifestyles and by building on public-private partnerships that can support access to healthy food for all community members.
