# Supplementary material for: Cardiovascular Disease in the Peruvian Andes: Local Perceptions, Barriers, and Paths to Preventing Chronic Diseases in the Cajamarca Region
Source: Int J Public Health. 2021 Sep 27;66:1604117. doi: 10.3389/ijph.2021.1604117 (PMC8504254; doi:10.3389/ijph.2021.1604117)
Supplement: Supplementary file 1 [file DataSheet1.pdf]

Cardiovascular Disease in the Peruvian Andes: Local Perceptions, Barriers, and Paths to Preventing Chronic Diseases in the Cajamarca Region

Giuliana Sanchez Samaniego 1,2,3

Stella Hartinger Peña 1,2,3

Paula Skye Tallman 4, 5

Daniel Mäusezahl 1,2

Affiliations:

1 Swiss Tropical and Public Health Institute, Basel, Switzerland

2 University of Basel, Basel, Switzerland

3 Universidad Peruana Cayetano Heredia, Lima Peru.

4 The Field Museum of Natural History, Chicago, IL, USA

5 Loyola University in Chicago, Chicago, IL, USA

Appendix 1. Example of principal questions of focus group discussion guideline.

This document presents a list of example of principal questions of the focus groups guideline used in eight focus groups in the provinces of San Marcos and Cajabamba. The guidelines was based in the Health Belief Model and additionally three sections were added: patterns of distress, perceived caused and gendered differences.

Patterns of distress/perceived causes
Which words come to mind when you think of cardiovascular diseases?
How would you know you have heart disease?
Can you tell me what could cause heart disease?

Perceived susceptibility
Do you think you can get a heart disease? Do you worry about getting cardiovascular diseases?
Where would you go if you started to feel unwell and thought it was due to your heart/ a cardiovascular disease?

Perceived severity
If your doctor diagnosed you with a heart disease... How would you feel?
How would having this disease change your life?
Do you worry about gaining or loosing too much weight? How would you feel if a doctor tells you to regulate your weight for health reasons?

Perceived benefits
What are the benefits of taking care of your heart?
What steps can you take to prevent a heart disease?
Do you enjoy physical activity? Do you consider that it brings you good health/benefits?

Barriers
Do you have access to information on and about heart disease?
Are you able to buy all the foods that you believe/consider to be good for your health?
Do you have time for physical activity?
What could prevent you from taking these measures, e.g. loose weight, do sports?

Self-efficacy

How confident are you that you can eat a balanced diet, with less carbohydrates and more protein and vegetables all-year round?

Cues to action

Do you think that trying new recipes could help you change your diet?

How do you feel about getting advice and receiving nutritional talks or going to group classes on healthy eating?

Gender

What happens in the lives of women in this community? Do you think they are at higher or lower risk of these diseases compared to men?
